# Nutzerverhalten bei der Funktionsprüfung des Beatmungsgerätes: Analyse zu Einsatz des KURZcheck und Detektion fehlkonnektierter Beatmungsschläuche

**DOI:** 10.1007/s00101-024-01496-0

**Published:** 2025-01-30

**Authors:** Davut D. Uzun, Johannes Schäfer, Sascha Klemm, Christoph Lichtenstern, Markus A. Weigand, Christopher Neuhaus

**Affiliations:** Medizinische Fakultät Heidelberg, Klinik für Anästhesiologie, Universität Heidelberg, Im Neuenheimer Feld 420, Heidelberg, Deutschland

**Keywords:** Anästhesiegerät, Schlauchverwechslung, Humanfaktoren, Fehlkonnektion, Patientensicherheit, Anesthesia workstation, Tube confusion, Human factors, Misconnection, Patient safety

## Abstract

**Hintergrund und Fragestellung:**

Die Patientensicherheit bei Allgemeinanästhesien hat sich in den letzten Jahren verbessert, dennoch kommt es immer wieder zu anästhesiologischen Zwischenfällen, insbesondere im Bereich der Atemwegssicherung. Vor dem Einsatz eines Beatmungsgerätes ist ein obligatorischer Systemtest erforderlich, zusätzlich sollte der von der DGAI empfohlene KURZcheck vor Anschluss eines Patienten durchgeführt werden. Dennoch sind Fehlanschlüsse der Beatmungsschläuche, die nicht zwingend vom Gerät im Rahmen des Selbsttests erkannt werden, möglich. Ziel der vorliegenden Studie war die Analyse des Nutzerverhaltens an modernen Anästhesiearbeitsplätzen im Hinblick auf den Einsatz des KURZcheck.

**Material und Methoden:**

Es erfolgten eine monozentrische, prospektive Beobachtung des Nutzerverhaltens im Umgang mit dem KURZcheck im Rahmen eines medizinischen Simulationstrainings sowie eine prospektive, multizentrische, explorative, anonyme Befragung des anästhesiologischen Personals in verschiedenen deutschen Kliniken.

**Ergebnisse:**

Von *n* = 30 beobachteten Probanden führten *n* = 28 (93,3 %) unmittelbar vor dem Anschluss des Simulationspatienten an das Beatmungsgerät einen KURZcheck durch. Nur einer dieser 28 erkannte die Fehlkonnektion der Beatmungsschläuche im Rahmen des KURZcheck. Den am Beatmungsgerät vorgehaltenen separaten Handbeatmungsbeutel nutzten trotz persistierender Beatmungsprobleme nur 20 % der teilnehmenden Ärzte. Die Online-Umfrage wurde von *n* = 187 Teilnehmenden beantwortet. 64,7 % der Teilnehmenden führen den KURZcheck nach eigenen Angaben immer durch, 31,5 % manchmal und 3,7 % nie. Das Vorhandensein eines separaten Handbeatmungsbeutels wird von 66,3 % der Befragten immer überprüft, von 29,8 % manchmal und von 3,8 % nie. 32 % der Ärzte waren der Meinung, dass der integrierte Selbsttest eine Fehlkonnektion der Beatmungsschläuche immer erkennt.

**Schlussfolgerung:**

Die vorliegende Studie zeigt ein heterogenes Bild der Anwendung und des Verständnisses des von der DGAI empfohlenen KURZcheck. Die in der Umfrage offenbarten Wissenslücken in Bezug auf Durchführung, Möglichkeiten und Grenzen des Checks unterstreichen die Bedeutung einer Ausbildung mit den Schwerpunkten Human Factors, Kommunikation und Kooperation im Gegensatz zu einer rein prozeduralen Umsetzung bewährter Werkzeuge.

**Graphic abstract:**

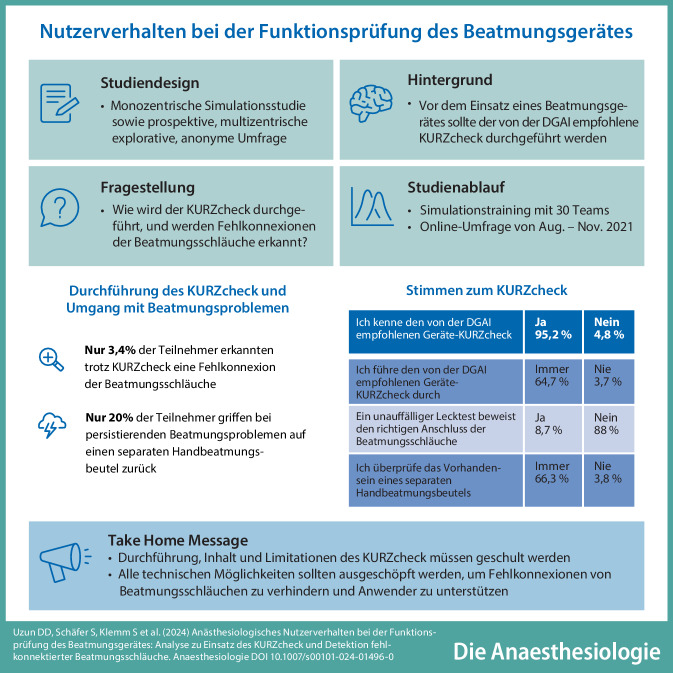

**Zusatzmaterial online:**

Die Online-Version dieses Artikels (10.1007/s00101-024-01496-0) enthält den Online-Fragebogen zum Gerätecheck: Bitte scannen Sie den QR-Code.

## Abstract (en)

**Background and research question:**

While patient safety during general anesthesia has improved in recent years, incidents still occur in anesthesia, particularly in the area of airway management and ventilation. In addition to a mandatory daily system test before using a ventilator, a QUICK check is recommended by the German Society of Anesthesiology and Intensive Care Medicine (DGAI) before connecting a patient; however, misconnections of breathing tubes are still possible and not necessarily detected by the device self-test. The aim of the present study was to analyze user behavior at modern anesthesia workstations regarding the verification and functionality of the QUICK check.

**Material and methods:**

A monocentric prospective recording of user behavior in handling the QUICKcheck was carried out as part of a medical simulation training course as well as a prospective, multicenter, explorative, anonymous survey of anesthesiology staff in various German hospitals.

**Results:**

Out of 30 participating physicians 93.3% (*n* = 28) carried out a QUICK check immediately before connecting the simulation patient to the ventilator. Only 1 (96.6%) of the physicians did not recognize the incorrect connection of the ventilation tubes during their QUICK check. Of the participating physicians, 80% did not use the separate bag valve mask despite persistent ventilation problems on the ventilator. The online survey was answered by *n* = 187 participants, 64.7% of the participants stated that they always carry out the QUICK check, 31.5% sometimes and 3.7% never. The presence of a separate bag valve mask is always checked by 66.3% of respondents, by 29.8% sometimes and by 3.8% never. Of the physicians, 32% believed that the integrated self-test always recognizes a misconnection of the ventilation tubes.

**Conclusion:**

The present study reveals a heterogeneous picture of the application and understanding of the QUICK check as recommended by the DGAI. The lack of understanding of a standardized implementation of the check can be seen as further evidence of the importance of interdisciplinary training with a focus on human factors, communication and cooperation as opposed to a mere procedural implementation of established tools.

Obgleich die Patientensicherheit im Rahmen von Allgemeinanästhesien kontinuierlich zunimmt, ereignen sich dennoch immer wieder Zwischenfälle. In diesen Fällen ist ein besonderes Augenmerk auf die Atemwegssicherung und die invasive Beatmung der Patienten zu richten. Vor dem Einsatz eines Beatmungsgerätes ist ein obligatorischer Systemtest erforderlich, zusätzlich sollte der von der Deutschen Gesellschaft für Anästhesiologie und Intensivmedizin (DGAI) empfohlene KURZcheck vor Anschluss eines Patienten durch den Anästhesiologen persönlich durchgeführt werden [17]. Dennoch kann es zu Fehlanschlüssen der Beatmungsschläuche kommen; diese werden nicht zwingend im Rahmen des Selbsttests vom Gerät erkannt und können mit fatalen Folgen einhergehen. Die vorliegende Studie hatte daher zum Ziel, das Nutzerverhalten an modernen Anästhesiearbeitsplätzen im Hinblick auf den Einsatz des KURZcheck zu analysieren.

## Hintergrund

Die Patientensicherheit ist im Rahmen von Allgemeinanästhesien in den letzten Jahren stetig gestiegen, trotzdem werden immer wieder anästhesiologische Zwischenfälle, teilweise mit letalem Ausgang, berichtet. In der Vielzahl dieser anästhesiologischen Zwischenfälle handelt es sich um Probleme oder Komplikationen im Bereich der Atemwegssicherung [15]. Die Funktionsprüfung des Narkosegerätes ist als ein wesentlicher Bestandteil der Strategie zur Vermeidung dieser Zwischenfälle in den letzten Jahren in den Fokus zahlreicher Publikationen, die sich insbesondere mit der Gewährleistung der Patientensicherheit befassen, gerückt [10, 17]. Dabei werden in hohem Maße sicherheitsrelevante Probleme im Zusammenhang mit der Beatmung von Patienten thematisiert.

Der moderne Anästhesiearbeitsplatz kann aus systemtheoretischer Betrachtung als komplexes soziotechnisches System aus mehreren Akteuren beschrieben werden. Die heutzutage verwendete Technik, beispielsweise in Form von Patienten-Monitoren oder Beatmungsgeräten, erweitert nicht nur die physischen Kapazitäten des Anästhesisten durch die Automatisierung von Handlungen (automatische vs. manuelle Blutdruckmessung oder Beatmung), sondern auch seine mentalen Kapazitäten (z. B. Darstellung und Integration einer Vielzahl von Parametern, Abnahme von Alarmierungs‑/Erinnerungsfunktion). Daher verfügen die Geräte über eigene Alarmierungs- und Handlungslogiken, die auf in das Design der Maschine integrierten Vorstellungen vom Verhalten des Nutzers basieren [5].

Die Bezeichnung „Joint Cognitive System (JCS)“ wird verwendet für eine Form der Verzahnung von Menschen und Maschinen, bei der alle beteiligten Komponenten Daten empfangen, analysieren und darauf basierend handeln können. Die Auswirkungen dieser Mensch-Maschine-Interaktion werden u. a. in der Domäne des Cognitive Systems Engineerings (CSE) erforscht [8]. Vor Inbetriebnahme eines Beatmungsgerätes sieht die Medizinprodukte-Betreiberverordnung (MPBetreibV) einen obligatorischen Systemtest des Beatmungsgerätes vor, bevor der Anschluss eines Patienten an das Gerät erfolgt. In diesem Kontext ist auch insbesondere der von der DGAI (zusätzlich zum von MPBetreibV geforderte Test) empfohlene KURZcheck zu nennen, bei dem durch den Anwender vor der Konnektion eines Patienten wichtige Komponenten des Beatmungsgerätes anhand einer Checkliste kursorisch überprüft werden [17]. Der KURZcheck umfasst drei essenzielle Aspekte; diese sind in Infobox 1 dargestellt [17]:

### Infobox 1 Anteile des Geräte KURZcheck. (Nach Prien et al. [17])

- Orientierend wird vor Anschluss des Patienten an das Beatmungsgerät die Gasfluss-Funktionalität des Atemsystems geprüft, dessen korrekte Montage, und ob große Leckagen bzw. Obstruktionen vorhanden sind („pressure and flow test, PaF“).
- Nach Anschluss des Patienten wird anhand der F_I_O_2_-Messung verifiziert, dass das farb- und geruchlose Gasgemisch, das dem Patienten zugeführt wird, genug Sauerstoff enthält.
- Nach Anschluss des Patienten wird mittels Kapnographie verifiziert, dass die Lungen ventiliert werden.

Auch nach Durchführung des Gerätesystemchecks gemäß MPBetreibV können noch unerkannte oder zwischenzeitlich neu aufgetretene Fehler vorliegen. Beispiele hierfür sind in Tab. 1 aufgeführt.

**Tab. 1:** „Wenn ein Patient an ein Narkosegerät angeschlossen wird, überprüfe ich/führe ich durch …“

Antwortmöglichkeiten	Immer (in %)	Manchmal (in %)	Nie (in %)	Gesamt *n*
… das Vorhandensein eines separaten Handbeatmungsbeutels	66,3 (*n* = 122)	29,8 (*n* = 55)	3,8 (7)	184
… einen „pressure and flow test“ vor Anschluss des Patienten	73,2 (*n* = 134)	23,5 (*n* = 43)	3,2 (*n* = 6)	183
… visuelle Kontrolle des korrekten Anschlusses der Beatmungsschläuche	74,7 (*n* = 136)	24,7 (*n* = 45)	0,5 (*n* = 1)	182
… eine Funktionsüberprüfung der Absaugung	69,4 (*n* = 127)	26,2 (*n* = 48)	4,3 (n8)	183
… den Füllungszustand der Vapore	29,6 (*n* = 54)	52,7 (*n* = 96)	17,5 (*n* = 32)	182
… einige manuelle Atemhübe vor Beginn der maschinellen Beatmung	63,5 (*n* = 117)	28,2 (*n* = 52)	8,1 (*n* = 15)	184
… den Sauerstofffluss (anhand der gemessenen F_I_O_2_)	78,2 (*n* = 144)	17,3 (*n* = 32)	4,35 (*n* = 8)	184
… ob etCO_2_ kommt (anhand Kapnographie/etCO_2_)	88,0 (*n* = 162)	9,2 (*n* = 17)	2,7 (*n* = 5)	183

### Merke.

**Gemäß aktueller Empfehlung der DGAI soll *immer***,** bevor ein Patient an ein Narkosegerät angeschlossen wird, ein KURZcheck durchgeführt werden** [17].

Die Konnektion der Beatmungsschläuche ist aufseiten des Gerätekonus und des Schlauchsystems durch die DIN-EN -SO-Norm 5356 genormt. Die durch diese Normierung begünstigten, potenziellen Fehlkonnektionen (z. B. zwischen In- oder Exspirationsschenkel und Anschluss für den Handbeatmungsbeutel) können durch den KURZcheck nicht verhindert, aber rechtzeitig entdeckt werden. Die Fehlkonnektion der Beatmungsschläuche stellt eine besondere Herausforderung und konsekutiv ein sicherheitsrelevantes Problem dar [16].

Zum jetzigen Zeitpunkt werden nicht alle Formen der Fehlkonnektion von Beatmungsschläuchen zuverlässig durch interne Kontrollmechanismen des Beatmungsgerätes, wie Systemchecks oder Hinweise/Alarme, erkannt. Die Relevanz der Thematik wird durch eine Vielzahl von anästhesiologischen Zwischenfällen, die teilweise mit Patientenschäden endeten, unterstrichen [17]. Auch dem Bundesinstitut für Arzneimittel und Medizinprodukte (BfArM) sind Ereignisse im Zusammenhang mit Fehlkonnektionen der Beatmungsschläuche über „Risikomeldungen“ bekannt geworden, was dieses zu einer Stellungnahme gemeinsam mit der Deutschen Gesellschaft für Anästhesiologie und Intensivmedizin (DGAI) bereits in früheren Jahren veranlasste [4]. In einigen der gemeldeten Fälle, die einen Patientenschaden zur Folge hatten, konnte der Fehler im Bereich der Fehlkonnektion der Beatmungsschläuche als Ursache identifiziert werden. Diese Fehlkonnektionen wurden entweder nicht oder zu spät durch die behandelnden Ärzte erkannt, was die suffiziente Beatmung und konsekutive Oxygenierung der Patienten verhinderte [4].

In den genannten Fällen wurde zu spät auf alternative Beatmungsmöglichkeiten wie z. B. einen Handbeatmungsbeutel zurückgegriffen. Diese Rückfallebene stellt jedoch einen der elementaren Bestandteile des von der DGAI empfohlenen Algorithmus für den Umgang mit beatmungsassoziierten Komplikationen dar [17]. Die zuvor genannten Handlungs- und Ablaufempfehlungen der DGAI sollten als integraler Bestandteil der anästhesiologischen Fort‑/Weiterbildung etabliert werden. Ihre regelmäßige Anwendung in Form von medizinischen Simulationstrainings gewährleistet dabei potenziell die Sicherstellung ihrer Anwendung in kritischen Situationen.

Das Ziel der vorliegenden Arbeit war eine Analyse des Nutzerverhaltens im Umgang mit modernen Narkosearbeitsplätzen im Hinblick auf die Überprüfung der Funktionsfähigkeit (Systemcheck oder KURZcheck) (Infobox 2). Es sollte evaluiert werden, ob derartige Überprüfungsverfahren unabhängig von ihrer theoretischen Sicherheitsfunktion mit Einfluss auf die Wirksamkeit unterworfen sind.

### Infobox 2 Exemplarische Fehler, die nach abgeschlossenem Gerätesystemcheck gemäß MPBetreibV trotzdem vorliegen können. (Modifiziert nach Prien et al. [17])

- Falsches Gas in der Sauerstoffleitung (z. B. durch Vertauschen der Leitungen im Gerät),
- Falsch gesteckte Atemschläuche (z. B. Kurzschluss zwischen Inspiration und Exspiration)
- Defekter bzw. fehlender Handbeatmungsbeutel
- Leckagen (z. B. Beatmungsschläuche, Wasserfallen, Handbeatmungsbeutel, CO_2_-Absorber)
- Verschlossener patientennaher Atemsystemfilter (z. B. Produktionsfehler oder ausgestanzte Verpackungsmaterialien im Lumen)
- Fehlender Atemkalkbehälter oder verbrauchter Atemkalk
- Defekter Atemkalkbehälter oder defekte Einrastvorrichtung, undichter Atemkalkbehälter
- Leckage im Bereich der Anästhesiemittelverdampfer, z. B. durch nichtverschlossene Füllöffnungen
- Anästhesiegasfortleitung nicht angeschlossen oder fehlerhaft

## Studiendesign und Untersuchungsmethoden

### Studiendesign

Es erfolgten eine monozentrische prospektive Erfassung des Nutzerverhaltens im Umgang mit dem KURZcheck im Rahmen eines medizinischen Simulationstrainings sowie eine prospektive, multizentrische explorative, anonyme Umfrage des anästhesiologischen Personals in verschiedenen deutschen Kliniken.

### Kollektiv

Im Rahmen des medizinischen Simulationstrainings konnten insgesamt *n* = 30 ärztliche Mitarbeitende der Klinik für Anästhesiologie des Universitätsklinikums Heidelberg in die Studie eingeschlossen werden.

Zur Teilnahme an der Online-Umfrage wurde ärztliches Personal aus der Anästhesiologie an vier Universitätskliniken und zwei Schwerpunktversorgern eingeladen (*n* = 800 Mitarbeitende).

### Einschlusskriterien

- Alter über 18 Jahre,
- Arzt in Weiterbildung oder Facharzt für Anästhesiologie.

### Ausschlusskriterien

- Ablehnung durch die Teilnehmer.

### Studienablauf

Die Fragestellung wurde in einem zweistufigen Prozess evaluiert. Im Rahmen der „Simulations-Woche“ der Klinik für Anästhesiologie des Universitätsklinikums Heidelberg wird allen ärztlichen und pflegerischen Mitarbeitenden der Klinik die Möglichkeit geboten, freiwillig an einem halbtägigen medizinischen Simulationstraining (bestehend aus mehreren ca. 30-minütigen Szenarien mit anschließendem Debriefing) am Heidelberger Anästhesie- und Notfallsimulationszentrum (HANS) teilzunehmen.

**Abb. 1:**
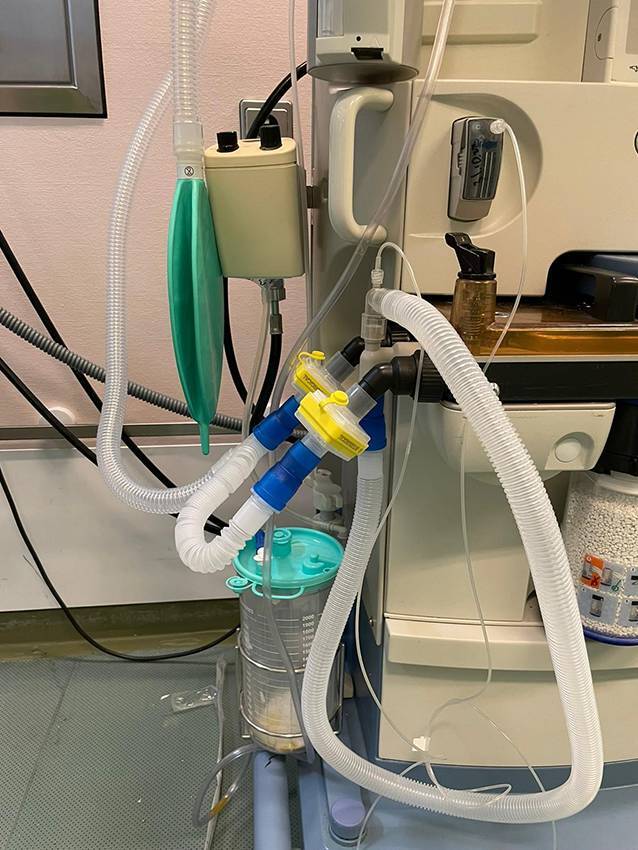
Fehlkonnektion der Beatmungsschläuche

Im Rahmen der Simulation wurden für das anschließende Debriefing anonyme Daten erfasst, um eine strukturierte Analyse und Nachbesprechung der stattgefundenen Ereignisse zu ermöglichen. Vom 11.03.2019 bis 15.03.2019 wurde eine wie oben beschriebene Fortbildungsveranstaltung (Simulationstraining) mit 30 Teams aus je einem ärztlichen und einem pflegerischen Teilnehmenden durchgeführt. Im Rahmen dieser Veranstaltung wurde ein Szenario, in dem eine Fehlkonnektion der Beatmungsschläuche am Narkosegerät simuliert wurde, präsentiert (Abb. 1). Der Umgang der Teilnehmer mit diesem Zwischenfall, insbesondere im Hinblick auf die Durchführung des KURZcheck, die Detektion der Fehlkonnektion bzw. das Management des daraus resultierenden Beatmungsproblems wurde durch eine Analyse der Debriefing-Datensätze erfasst. Nach Exklusion der unvollständigen Datensätze konnten in die endgültige Analyse 30 teilnehmende Ärztinnen und Ärzte aufgenommen werden.

**Abb. 2:**
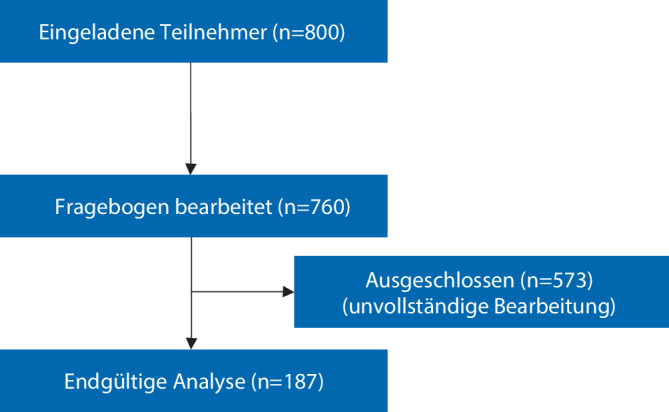
Flussdiagramm zum Studieneinschluss gemäß STROBE-Statement

Im zweiten Schritt erfolgte vom 25.08.2021–07.11.2021 eine multizentrische anonyme Online-Umfrage unter ärztlichen Mitarbeitenden, welche deren Verhalten im Alltag in Bezug auf die Überprüfung des Narkosearbeitsplatzes thematisierte. Die Umfrage erfolgte mit der Online-Plattform EFS Survey (www.unipark.de, Fa. Tivian XI GmbH, Köln). Der Zugang zur Umfrage erfolgte über einen nichtpersonalisierten Hyperlink. Den Einschluss der Teilnehmer zeigt Abb. 2.

### Statistik

Die erhobenen Daten, die der Auswertung zugrunde liegen, wurden mithilfe von deskriptiver Statistik analysiert. Diese erfolgte unter Angabe von absoluten und relativen Häufigkeiten bzw. deren Mittelwert und Standardabweichung. Die Analysen werden mit den Programmen SPSS und Microsoft Excel durchgeführt.

### Probandeninformation/Einverständniserklärung

Die Studie wurde gemäß der Deklaration von Helsinki in der jeweils gültigen Fassung durchgeführt. Das Studienprotokoll wurde durch die Ethikkommission der Medizinischen Fakultät Heidelberg positiv begutachtet (S-518/2019). Die vorliegende Studie erfüllt die Anforderungen des STROBE-Statements [18].

## Ergebnisse

### Simulierte Fehlkonnektion der Beatmungsschläuche

Das Teilnehmerkollektiv des Simulationstrainings setzte sich zu 33,3 % (*n* = 10) aus Fachärzten und zu 66,7 % (*n* = 20) aus Ärzten in Weiterbildung zusammen. Zum Zeitpunkt der Untersuchung befanden sich die Ärzte in Weiterbildung im Median im dritten Weiterbildungsjahr zum Facharzt für Anästhesiologie. Von den insgesamt 30 ärztlichen Teilnehmenden führten 93,3 % (*n* = 28) unmittelbar vor dem Anschluss des Simulationspatienten an das Beatmungsgerät einen KURZcheck durch, 6,7 % (*n* = 2) der Teilnehmenden führten *keinen* KURZcheck durch. Beide Teilnehmer, die keinen KURZcheck durchführten, waren Teilnehmer aus der Gruppe der Fachärzte.

### Detektion von Fehlkonnektion mittels KURZcheck

96,6 % der teilnehmenden Ärzte entdeckten die Fehlkonnektion der Beatmungsschläuche im Rahmen ihres KURZcheck nicht. Bei den teilnehmenden Ärzten, die den Fehler im Rahmen des KURZcheck erkannt haben, handelt es sich ausnahmslos um fachärztliche Teilnehmer.

### Nutzung des separaten Handbeatmungsbeutels als „Back‑up“

**Abb. 3:**
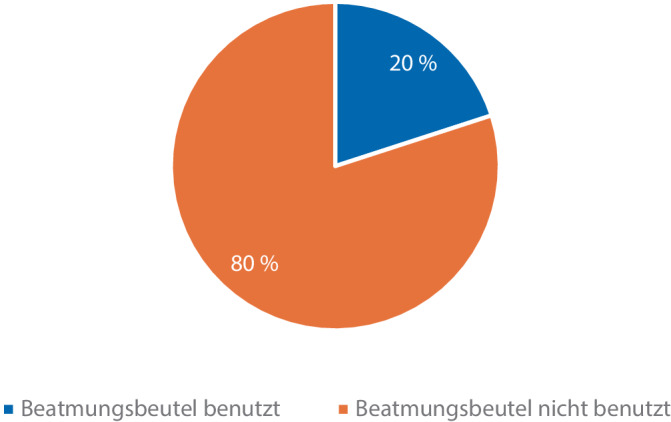
Anwendung des separaten Beatmungsbeutels bei persistierendem Beatmungsproblem

Es wurde weiterhin beobachtet, ob und in welchem Umfang Ärzte bei persistierenden Beatmungsproblemen am Beatmungsgerät den verfügbaren separaten Handbeatmungsbeutel anwenden. Insgesamt haben lediglich 20 % der teilnehmenden Ärzte diese Option gewählt. In der vorliegenden Simulationsstudie wurde somit beobachtet, dass 80 % der teilnehmenden Ärzte den separaten Handbeatmungsbeutel trotz persistierendem Beatmungsproblem am Beatmungsgerät nicht anwendeten (Abb. 3). Die Verteilung der Fachärzte und Ärzte in Weiterbildung beträgt 66,7 % zu 33,3 %.

## Ergebnisse der Online-Umfrage

Die multizentrische Online-Umfrage wurde von *n* = 760 ärztlichen Mitarbeiterinnen und Mitarbeitern aus verschiedenen deutschen anästhesiologischen Kliniken unterschiedlicher Versorgungsstufen begonnen; *n* = 187 Teilnehmer schlossen den Fragebogen vollständig ab und konnten in die Auswertung einbezogen werden (vollständiger Fragebogen im Zusatzmaterial online). Die Details zum Einschluss der Teilnehmer zeigt die Abb. 2. Eine Übersicht über die beruflichen Positionen der Teilnehmer liefert Tab. 2.

**Tab. 2:** Verteilung der beruflichen Positionen der Teilnehmenden

Chefarzt	*n* = 3 (1,6 %)
Oberarzt	*n* = 30 (15,9 %)
Facharzt	*n* = 52 (27,6 %)
Arzt in Weiterbildung	*n* = 103 (54,7 %)

95,2 % (*n* = 178) der Teilnehmenden gaben an, den DGAI-KURZcheck zu kennen; nur 4,8 % (*n* = 9) war dieser unbekannt.

Zur regelmäßigen Durchführung des von der DGAI empfohlenen KURZcheck gaben 64,7 % (*n* = 121) der Teilnehmenden an, den Check immer durchzuführen. 31,5 % (*n* = 59) beantworteten die Frage mit „manchmal“ und 3,7 % (*n* = 7) mit „nie“. Die Abfrage, inwiefern einzelne Items des Checks durchgeführt werden, ergab ein heterogenes Bild (Tab. 2).

32,0 % (*n* = 59) der Befragten gingen davon aus, dass der integrierte Selbsttest eines modernen Anästhesiegerätes den richtigen Anschluss der Beatmungsschläuche und des Handbeatmungsbeutels umfasst. 55,9 % (*n* = 103) beantworteten dies mit „nein“, 11,9 % (*n* = 22) der Befragten waren sich unsicher.

Die Frage danach, ob ein unauffälliger Lecktest den richtigen Anschluss der Beatmungsschläuche und des Handbeatmungsbeutels beweist, wurde von 8,7 % (*n* = 16) der Teilnehmer mit ja beantwortet. 88,0 % (*n* = 162) beantworteten die Frage mit nein, 3,2 % (*n* = 6) waren sich unsicher. Ähnliche Ergebnisse lieferte auch die Frage nach dem korrekten Vorgehen nach einem Schlauchsystemwechsel: 21,9 % (*n* = 40) der Teilnehmenden gaben an, dass nach einem Schlauchwechsel zwischen zwei Anästhesien eine Sichtprüfung auf korrekte Montage des Beatmungsschlauchsystems und ein Leckagetest ausreichen; 73 % (*n* = 133) verneinten dies, 4,9 % (*n* = 9) waren sich unsicher.

## Diskussion

Das Nutzerverhalten, insbesondere von prozeduralen Handlungsabläufen wie z. B. Gerätechecks in der Anästhesiologie kann mittels medizinscher Simulation suffizient erfasst, bewertet und trainiert werden. Die Wichtigkeit des von der DGAI empfohlenen KURZcheck wurde bereits in der Einleitung thematisiert; in unserer Studie führten bei einer simulierten Narkoseeinleitung 93,3 % (*n* = 28) der Teilnehmenden unmittelbar vor dem Anschluss des Simulationspatienten an das Beatmungsgerät diesen KURZcheck durch. Umso bemerkenswerter ist daher, dass trotz durchgeführtem KURZcheck nur einer von 28 Simulationsteilnehmern die Fehlkonnektion der Beatmungsschläuche bemerkt hatte. Angesichts der potenziell weitreichenden Folgen einer nichterkannten Fehlkonnektion von Beatmungsschläuchen ist dieses Ergebnis besorgniserregend und wirft Fragen zur Wirksamkeit derartiger Checks als gängige Sicherheitswerkzeuge auf. Bei bestehenden Beatmungsproblemen wird in den Handlungsempfehlungen der DGAI die Anwendung eines separaten Handbeatmungsbeutels empfohlen, um schnell zwischen patienten- und geräteseitigem Problem differenzieren zu können und eine möglichst zeitnahe Lösung des Beatmungsproblems zu erzielen [17]. In der vorliegenden Studie wendeten lediglich 20 % der teilnehmenden Ärzte diese empfohlene Maßnahme trotz persistierenden Beatmungsproblem an. Ärzte in Weiterbildung nutzten den separaten Beatmungsbeutel häufiger als fachärztliche Kollegen.

Allerdings scheint eine prozedurale („unbedachte“) Durchführung des KURZcheck ohne bewusste mentale Fokussierung auf dessen inhaltliche Bedeutung nur einen begrenzten Effekt auf die Vermeidung von Zwischenfällen zu haben. Nur so ist zu erklären, dass die Fehlkonnektion der Beatmungsschläuche nicht erkannt wurde. Bei einer konsequenten, bewussten Nachverfolgung der Schlauchsysteme mit der Hand – von geräteseitiger Konnektion bis zum Handbeatmungsbeutel bzw. Winkelstück – würde man eine Detektion der Fehlkonnektion erwarten. Somit scheint eine rein kursorische Durchführung des KURZcheck mit lediglich visueller Kontrolle der geräteseitigen Konnektionsstellen nur einen begrenzten Sicherheitsgewinn zu bieten. Hier zeigen sich erschreckende Parallelen zu einer Studie von Münsterer et al., die untersuchte, ob absichtlich eingebaute Fehler während des Team-Time-Out von den Mitgliedern des Operationsteams erkannt werden. Dabei wurden bei 1800 Operationen während des Team-Time-Out durch den Chirurgen Fehler bewusst eingebaut (z. B. Nennung des falschen Patientennamens, falsche Operationsseite etc.), die insgesamt nur in 54 % der Fälle durch die restlichen Teilnehmer des Time-Out bemerkt wurden. Am häufigsten wurden diese simulierten Fehler von Anästhesisten bemerkt (64 %). Auch hier zeigt sich also, dass bei „unbedachter“, prozeduraler Einhaltung und Abarbeitung der Time-Out-Richtlinien relevante Fehler übersehen werden können. Trotz bester Intention wird das Werkzeug Team-Time-Out auf ein – zugegebenermaßen für den Außenstehenden dramaturgisch eindrückliches – aber inhaltliches Lippenbekenntnis reduziert. Diesen Ergebnissen liegen verschiedene Einflüsse zugrunde: Themen wie Zeitdruck und inhaltliche Unkenntnis scheinen eine Rolle zu spielen, derartige Werkzeuge können aber auch als eine weitere von vielen bürokratischen Bürden empfunden werden statt als potenziell nützlich für Patientensicherheit und Teamdynamik. Als Konsequenz entspricht die Durchführung mehr einer routinierten „Litanei“ als eine aktiven, reflektierten Auseinandersetzung mit den Inhalten [11, 12]. Insbesondere jüngeren Kolleginnen und Kollegen ist, häufig aufgrund mangelnder Schulungsmaßnahmen, die Schlüsselposition derartig simpler, aber effektiver Werkzeuge wie eines korrekt und aufmerksam durchgeführten, fokussierten KURZcheck zur Gewährleistung der Patientensicherheit nicht bewusst. Auch der fehlende Griff zum Handbeatmungsbeutel kann mutmaßlich auf unterschiedliche mentale Modelle in der Handhabung scheinbar „unerklärlicher“ Beatmungsprobleme zurückgeführt werden und wirft die Frage auf, inwiefern derartige Rettungs- und Problemlösungsstrategien suffizient in der Weiterbildung gelehrt werden.

### Strukturelle Gründe

Obgleich weltweit eine hohe Dunkelziffer an Beatmungszwischenfällen zu vermuten ist, finden sich in der Literatur immer wieder Hinweise auf die Problematik, die aus dem Spannungsfeld zwischen Mensch und Maschine entsteht. Bereits im Jahr 2013 wies das BfArM auf die Wichtigkeit der Aspekte Usability und Ergonomie im Zusammenhang mit Medizinprodukten gemäß DIN EN 62366 hin [7]. Der Beitrag konstruktiver Änderungen an Medizinprodukten zur Patientensicherheit kann anhand von Beispielen aus der Vergangenheit verdeutlicht werden. Exemplarisch hierfür sind z. B. Luer-Lock-Konnektoren: Nach einer Häufung an dokumentierten Fehlverbindungen mit Todesfolge wurden diese durch die WHO als Patientenrisiko anerkannt. Daraufhin wurde die Normenreihe ISO 80369 mit verschiedenen Verbindertypen entwickelt [16]. Das angeführte Beispiel veranschaulicht jedoch auch die Schwierigkeiten, die mit derartigen Bemühungen einhergehen. Während bereits im Jahr 1968 Probleme mit Luer-Verbindungen dokumentiert wurden, ist die Medizin 50 Jahre später noch weit von einer konsequenten Umstellung entfernt [13]. Mit der neuen ISO 80601-2-13 gibt es seit Kurzem erstmalig geräteseitige Änderungen, die eine Fehlkonnektion von Beatmungsschläuchen unmöglich machen. Es ist allerdings davon auszugehen, dass bis zu einer flächendeckenden Anwendung der neuen Konnektoren noch viele Jahre bis Jahrzehnte vergehen. Unabhängig von technischen Lösungen muss somit weiterhin das menschliche Verhalten in komplexen Systemen reflektiert und trainiert werden.

### Online-Umfrage

Die im Anschluss an die Simulation durchgeführte, multizentrischen Online-Umfrage kann potenziell dazu beitragen, verschiedene mentale Modelle von Anästhesistinnen und Anästhesisten im Umgang mit bzw. im Vertrauen auf Beatmungsgeräte(n) im Kontext eines JCS aus Mensch und Maschine besser zu verstehen. Bei der Mehrzahl der Teilnehmenden handelte es sich um Ärzte in Weiterbildung und Fachärzte für Anästhesiologie. Da Ober- und Chefärzte in der Regel eine Supervisionsrolle einnehmen und für mehrere OP verantwortlich sind, lässt sich mutmaßen, dass die Anzahl der durchgeführten Gerätechecks pro Tag bei Ärzten in Weiterbildung und Fachärzten höher ist als bei Ober- und Chefärzten.

Gemäß § 4 Abs. 6 MPBetreibV (Medizinprodukte-Betreiberverordnung) ist eine Prüfung der Funktionsfähigkeit und des ordnungsgemäßen Gerätezustandes vor Anwendung verbindlich vorgeschrieben. Die hier vorliegende Studie demonstriert die weitläufige Bekanntheit des KURZcheck unter den befragten Ärztinnen und Ärzten: In der vorliegenden Befragung war dem Großteil (95 %) der ärztlichen Mitarbeitenden der von der DGAI empfohlene KURZcheck bekannt. Die Empfehlung zur Durchführung basiert auf der Prämisse, dass neben der obligatorischen, durch die MPBetreibV geforderten, vollständigen Funktionsprüfung eines Narkosegeräts ein KURZcheck vor/bei Anschluss eines Patienten an dieses durchgeführt werden sollte. Dieser KURZcheck umfasst die Prüfung der wichtigsten Funktionen und ermöglicht somit eine effiziente und zeitnahe Überprüfung des Geräts auch in Notfallsituationen, was die Patientensicherheit maßgeblich erhöhen kann [2]. So untersuchten Arbous et al. die Risikofaktoren des anästhesiologischen Managements in Bezug auf die postoperative 24-h-Morbidität und 24-h-Mortalität bei 869.483 Patienten. In dieser Fall-Kontroll-Studie konnte ein signifikant reduziertes Risiko bei Durchführung einer standardisierten Prüfung der anästhesiologischen Gerätschaften anhand einer Checkliste oder eines Protokolls identifiziert werden (OR 0,64) [1, 2]. Diese Ergebnisse bekräftigen die Erkenntnis, dass eine Prüfung der Anästhesiegeräte durch den Anästhesisten persönlich ein Instrument zur Risikominimierung im Rahmen der Narkose darstellt. Analysen aus anästhesiologischen Zwischenfallmeldesystemen zeigen, dass ca. ein Viertel der „technischen“ Beatmungsprobleme auf einen insuffizienten Gerätecheck zurückzuführen ist. Ferner scheinen weitere 25 % der berichteten Zwischenfälle im Bereich der Beatmungsschläuche sowie Beatmungsfilter zu liegen [17]. Obgleich die Relevanz des KURZcheck evident ist, zeigte sich in unserer Evaluation bei 4,8 % der Teilnehmenden keine Kenntnis über den von der DGAI empfohlenen Gerätecheck.

Laut eigenen Angaben der Teilnehmenden führten lediglich 64 % der Ärzte den KURZcheck, wie von der DGAI gefordert, „immer“ durch. 31 % der Befragten gaben an, den Check unregelmäßig, also manchmal, durchzuführen. Prien et al. führen in ihren Empfehlungen der Kommission für Normung und technische Sicherheit der DGAI sehr detailliert auf, welche Gefahren durch ein Nichtbeachten der Empfehlungen hinsichtlich des KURZcheck drohen, und welche Probleme durch die geräteeigenen Systemchecks nicht erkannt werden [17]. Folglich liegt erneut nahe, dass ein relevanter Mangel an Schulung und Fortbildung des anästhesiologischen Personals in Bezug auf dieses sensible Thema vorherrscht; dies erklärt potenziell, wieso der eigentlich obligatorisch vorgeschriebene Check nur in so geringer Häufigkeit durch die Ärztinnen und Ärzte durchgeführt wird. Da in der Weiterbildungsordnung nur globale „Maßnahmen der Qualitätssicherung und des Qualitätsmanagements einschließlich des Fehler- und Risikomanagements“ als Ausbildungsziel beschrieben sind, aber keine detailliertere Aufschlüsselung der Lerninhalte, insbesondere für den Umgang mit Beatmungsgeräten, formuliert ist, kann nicht davon ausgegangen werden, dass Fachärzte nur aufgrund Ihrer FA-Qualifikation für diesen Bereich hinreichend sensibilisiert sind. Es sollten in Zukunft weiterhin Anstrengungen unternommen werden, um eine möglichst hohe KURZcheck-Rate im Sinne der Patientensicherheit in der Anästhesiologie zu erreichen. Die fehlende Durchführung des KURZcheck kann darüber hinaus, nicht zuletzt aufgrund der Position der DGAI, auch im Rahmen der Verantwortungszuweisung nach Zwischenfällen eine potenziell rechtliche Relevanz bekommen [17].

In der vorliegenden Studie gaben lediglich 66 % der Befragten an, bei der Überprüfung des Anästhesiearbeitsplatzes das Vorhandensein eines separaten Handbeatmungsbeutels zu überprüfen. In den von der DGAI publizierten Handlungsempfehlungen wird zu systematischen Fehlersuche bei Stenosen im Atemsystem eine sofortige Beatmung mit einem separaten Beatmungsbeutel (ohne Atemsystemfilter) empfohlen [17]. Daher ist es umso überraschender, dass die Überprüfung, ob ein separater Beatmungsbeutel zur Verfügung steht, nicht regelhaft stattfindet. Falls die Beatmung mittels separatem Beatmungsbeutel leicht möglich ist, ist die Stenose mit hoher Wahrscheinlichkeit im Bereich des Atemsystemfilters, der Beatmungsschläuche oder am Beatmungsgerät lokalisiert.

Hingegen scheint durch die befragten Ärzte die Überprüfung des etCO_2_ nach Anschluss eines Patienten an das Beatmungsgerät in nahezu 90 % der Fälle zu erfolgen.

In unserer Befragung gaben 69 % der Ärztinnen und Ärzte an, regelhaft die Funktionsprüfung der Absaugung durchzuführen. Die pulmonale Aspiration ist eine gefürchtete Komplikation jeder Allgemeinanästhesie. Im Falle einer Regurgitation ist zur schnellen und sicheren Dekontamination des Atemwegs eine funktionsfähige Absaugeinheit am Anästhesiearbeitsplatz unerlässlich. Über 30 % der Befragten führen laut eigenen Angaben keinen Check der Absaugeinheit durch. Dieser Umstand sollte ebenfalls im Rahmen von strukturieren Trainingsprogrammen benannt und, wie von der DGAI empfohlen, in den Standardablauf inkludiert werden [17].

Zur Frage der Verlässlichkeit hinsichtlich des integrierten Selbsttests eines modernen Narkosegerätes sowie der richtigen Anschlüsse der Beatmungsschläuche und des Handbeatmungsbeutels gaben 32 % der Teilnehmenden an, dass der integrierte Geräteselbsttest eine Fehlkonnektion der Beatmungsschläuche „entdeckt“. 22 % der Teilnehmenden waren sich in dieser Thematik nicht sicher. Hieraus lässt sich ableiten, dass einem relevanten Anteil der Befragten die Problematik der Fehlkonnektion der Beatmungsschläuche trotz durchgelaufenen Selbsttests des Beatmungsgerätes nicht bekannt war. Daher besteht auch in diesem Bereich noch weiterer Fortbildungsbedarf. Diesbezüglich scheint es empfehlenswert, entsprechende Schulungen im Rahmen der Herstellereinweisungen in die Beatmungsgeräte oder im Rahmen von regelmäßigen „Auffrischungen“ der Einweisungen oder in strukturierten Trainings anzubieten.

### Limitationen

Die Rücklaufquote der Befragung betrug rund 23 % und stellt somit im Vergleich zu vergleichbaren Umfragen ein befriedigendes Ergebnis dar. Mögliche Hindernisse für eine höhere Beteiligung können die begrenzte Dauer der Befragung sowie der zeitliche Aufwand für die Teilnehmer darstellen. Des Weiteren wurden keine monetären oder sonstigen Anreize für die Teilnehmer bereitgestellt [6]. Die Anonymität der Online-Befragung führt dazu, dass soziale Erwünschtheit nur eine untergeordnete Rolle spielt, wodurch diese anderen Befragungsformaten überlegen ist [9]. Die Stärke der vorliegenden Befragung liegt in der gezielten Auswahl der Adressaten, welche eine homogene Stichprobe mit für das Thema relevanten Teilnehmern generiert [3, 14].

Aufgrund des Studiendesigns war allen Teilnehmenden der Simulation bewusst, dass sie im Rahmen des Trainings unter Beobachtung standen. Dies kann zu einer Verzerrung der Ergebnisse führen. Leider ermöglicht das Studiendesign keine abschließende Klärung der Frage, warum die Schlauchverwechslung „übersehen“ wurde: Die Teilnehmenden zeigten sich in den Debriefings – insbesondere in Anbetracht der klinischen Relevanz und Dramatik im realen Fall – zwar über ihr eigenes Verhalten erstaunt, verärgert oder sogar beängstigt. Befriedigende Erklärungen für das eigene Handeln konnten allerdings nicht genannt werden, vielmehr überwogen Reaktionen wie Sprachlosigkeit, Verwunderung oder Entsetzen. Dennoch wurde durch die Teilnehmenden die Durchführung derartiger Simulationstrainings begrüßt.

## Schlussfolgerung

Die durchgeführte Studie zeigt ein heterogenes Bild hinsichtlich der inhaltlich sinnvollen Anwendung des von der DGAI empfohlenen KURZcheck. Je nach Art der Durchführung bzw. dabei bewusst gelenkter Aufmerksamkeit scheint der KURZcheck bei der Erkennung von Fehlanschlüssen von Beatmungsschläuchen nur bedingt erfolgreich zu sein. Strukturierte und regelmäßig wiederholte Schulungsmaßnahmen (z. B. Simulationstrainings) könnten eine effektive Methode zur Erlangung und Aufrechterhaltung der Kompetenzen in diesem Bereich darstellen. Darüber hinaus sollten weiterhin alle technischen und konstruktiven Möglichkeiten ausgeschöpft werden, um Fehlkonnektionen von Beatmungsschläuchen zu verhindern und Anwender bei der Risikominimierung und Durchführung sicherer Narkosen zu unterstützen.

## Fazit für die Praxis

- Der von der DGAI empfohlene KURZcheck scheint seinen Beitrag zur Gewährleistung der Patientensicherheit nur leisten zu können, wenn Durchführung, Inhalt und Limitationen fundiert und regelmäßig geschult werden.
- Es sollten weiterhin alle technischen und konstruktiven Möglichkeiten ausgeschöpft werden, um Fehlkonnektionen von Beatmungsschläuchen zu verhindern und Anwender bei der Risikominimierung und Durchführung sicherer Narkosen zu unterstützen.
- Bei Problemen bei der Beatmung sollten die Anwender zum separaten Handbeatmungsbeutel (ohne Filter) greifen.
- Algorithmen zur Fehlersuche bei „Leckage“- bzw. „Stenose“-Symptomatik sollten regelmäßig im Rahmen von medizinischen Simulationen beübt werden.

## Supplementary Information

Fragebogen Gerätecheck

## Data Availability

Die in dieser Studie erhobenen Datensätze können auf begründete Anfrage beim Korrespondenzautor angefordert werden.
