# Supplementary material for: User behavior during functional testing of the ventilator: analysis of the use of the QUICKcheck and detection of misconnected ventilation tubes
Source: Anaesthesiologie. 2025 Jan 30;74(2):72–80. [Article in German] doi: 10.1007/s00101-024-01496-0 (PMC11835972; doi:10.1007/s00101-024-01496-0)
Supplement: Supplementary file 1 — Fragebogen Gerätecheck [file 101_2024_1496_MOESM1_ESM.pdf]

Zusatzmaterial zum Beitrag „Anästhesiologisches Nutzerverhalten bei der Funktionsprüfung des Beatmungsgerätes: Analyse zu Einsatz des KURZcheck und Detektion fehlkonnektierter Beatmungsschläuche“ von Uzun DD, Schäfer J, Klemm S et al. (2024) in D*ie Anaesthesiologie*.

Beitrag und Zusatzmaterial stehen Ihnen auf www.springermedizin.de zur Verfügung. Bitte geben Sie dort den Beitragstitel in die Suche ein.

Online - Fragebogen

Möglichkeiten und Grenzen des „Kurzchecks“ zur Funktionsprüfung des Narkosegerätes.

Version 1.1

Sehr geehrte Teilnehmerin, sehr geehrter Teilnehmer,

wir führen eine anonyme Umfrage zum Nutzerverhalten im Umgang mit Narkosegeräten durch. Hierfür würden wir Ihnen gerne einige Fragen stellen; die Beantwortung wird ca. 5 Minuten Zeit in Anspruch nehmen.

Wir erfassen dabei **keine** personenbezogenen Daten, Ihre Teilnahme ist **komplett anonym**. Die Teilnahme an der Studie erfolgt freiwillig. Falls Sie teilnehmen möchten, bitten wir Sie, die folgende Umfrage bis zu Ende zu beantworten; ein Abbruch ist jederzeit möglich. Inkomplett ausgefüllte Fragebögen werden nicht ausgewertet, erst mit der vollständigen Bearbeitung des Fragebogens erklären Sie Ihre Einwilligung. Die erhobenen Daten werden ausschließlich zu Zwecken dieser Studie verwendet. Die Studienleitung wird alle angemessenen Schritte unternehmen, um den Schutz der Daten gemäß den Datenschutzstandards der Europäischen Union zu gewährleisten. Die Daten sind gegen unbefugten Zugriff gesichert und werden für 5 Jahre aufgehoben. Ihnen entstehen aus der Teilnahme weder persönlicher Nutzen noch Risiken.

Die Umfrage wurde der Ethikkommission der Medizinischen Fakultät der Universität Heidelberg vorgelegt und erhielt ein positives Votum (Aktenzeichen S-518/2019).

Ich bin...

Chefarzt/Oberarzt/Facharzt/Assistenzarzt für Anästhesie

Berufserfahrung in Jahren (in der Anästhesie)

Bitte bewerten sie die folgenden Aussagen

1. Ich kenne den von der DGAI empfohlenen Geräte-KURZcheck
   (Antwortmöglichkeiten „ja", „nein")

2. Ich führe den von der DGAI empfohlenen Geräte-KURZcheck durch
   (Antwortmöglichkeiten „immer", „manchmal", „nie")

3. Wenn ein Patient an ein Narkosegerät angeschlossen wird, überprüfe ich/führe ich durch:

a) Das Vorhandensein eines separaten Handbeatmungsbeutels
b) Pressure and Flow Test vor Anschluss des Patienten
c) Visuelle Kontrolle des korrekten Anschlusses der Beatmungsschläuche
d) Funktionsüberprüfung der Absaugung
e) Füllungszustand der Vapore
f) Einige manuelle Atemhübe vor Beginn der maschinellen Beatmung
g) Sauerstofffluss anhand der gemessenen FiO2
h) Ob CO2 kommt (anhand Kapnographie/etCO2)
   (Antwortmöglichkeiten „immer", „manchmal", „nie")

4. Bei technischen Zwischenfällen während der maschinellen Beatmung liegt die Ursache häufiger
   a) Im Bereich der Beatmungsschläuche
   b) Im Bereich der Narkosegeräte

5. Der integrierte Selbsttest eines modernen Narkosegerätes umfasst den richtigen Anschluss der Beatmungsschläuche und des Handbeatmungsbeutels
   (Antwortmöglichkeiten „ja", „weiß nicht", „nein")

6. Ein unauffälliger Lecktest beweist den richtigen Anschluss der Beatmungsschläuche und des Handbeatmungsbeutels
   (Antwortmöglichkeiten „ja", „weiß nicht", „nein")

7. Nach einem Schlauchwechsel zwischen zwei Anästhesien genügt eine Sichtprüfung auf korrekte Montage des Schlauchsystems und ein Leckagetest
   (Antwortmöglichkeiten „ja", „weiß nicht", „nein")

Herzlichen Dank für Ihre Unterstützung!
